# CAR-T Cell Therapy for Prostate Cancer: Current Advances and Future Perspectives

**DOI:** 10.3390/biomedicines13102545

**Published:** 2025-10-18

**Authors:** Maria Luisa Calabrò, Roberta Ettari, Carla Di Chio, Fabiola De Luca, Santo Previti, Maria Zappalà

**Affiliations:** Department of Chemical, Biological, Pharmaceutical and Environmental Sciences, University of Messina, Viale Stagno d’Alcontres 31, 98166 Messina, Italy; mlcalabro@unime.it (M.L.C.); rettari@unime.it (R.E.); cdichio@unime.it (C.D.C.); fabiola.deluca@unime.it (F.D.L.); spreviti@unime.it (S.P.)

**Keywords:** chimeric antigen receptor, CAR-T cells, immunotherapy, prostate cancer, targeted prostate antigens

## Abstract

Prostate cancer is the most frequently diagnosed solid-organ malignancy in men worldwide. Metastatic castration-resistant prostate cancer represents a rapidly fatal, end-stage form of the disease for which current therapies remain palliative rather than curative. The advent of chimeric antigen receptor (CAR) T-cell therapy has revolutionized the treatment of refractory hematologic malignancies, and a growing number of studies are now exploring its potential in solid tumors. In this review, we first provide a concise overview of current immunotherapeutic strategies for prostate cancer, including checkpoint inhibitors, vaccine-based approaches, and bispecific antibodies. We then focus on the most recent and promising developments in CAR-T cell therapy for this malignancy. Specifically, we examine the key tumor-associated antigens targeted in prostate cancer-directed CAR-T cell therapy and summarize findings from preclinical research as well as ongoing and completed clinical trials. Finally, we discuss the main challenges that limit the efficacy of CAR-T therapy in prostate cancer, such as antigen heterogeneity, immunosuppressive tumor microenvironments, on-target/off-tumor toxicity, limited T-cell persistence, and inefficient trafficking to metastatic lesions, and outline potential strategies to overcome these barriers. Our aim is to define a translational roadmap for advancing CAR-T therapy toward clinical application in patients with metastatic castration-resistant prostate cancer.

## 1. Introduction

Prostate cancer (PCa) is the most common malignancy of the male genitourinary system and the fifth leading cause of cancer death among men worldwide [1,2]. In 2022, more than 1,460,000 estimated cases and 396,000 deaths were estimated [3]. PCa usually grows slowly and remains confined to the prostate gland, with a 5-year survival rate exceeding 95% [4]. However, there are also more aggressive forms, in which the diseased cells quickly invade nearby tissues and spread to distant organs [5]. Age is the main risk factor, as about 60% of prostate cancers are diagnosed in men over 65. Other risk factors include ethnicity, with African American and South American men showing the highest incidence rates, family history of the disease, and incorrect lifestyle (i.e., smoking, obesity, alcohol consumption) [6].

Globally, prostate cancer incidence and mortality show marked geographic and ethnic disparities. The highest age-standardized incidence rates occur in Northern Europe, North America, and Australia/New Zealand, primarily reflecting widespread PSA screening. On the contrary, mortality is disproportionately higher in sub-Saharan Africa and the Latin America/Caribbean, where access to early detection and treatment is limited. These inequalities underline the need for region-specific prevention and management strategies to reduce the global burden of disease [7].

PCa mainly originates from basal or luminal epithelial cells of prostatic tissue. In physiological conditions, growth and differentiation of prostate cells are finely regulated, mainly controlled by androgens. During prostatic carcinogenesis, anomalies in androgen receptor signaling may lead to abnormal growth and reduced apoptosis, contributing to disease development and progression. Furthermore, even in advanced stages of PCa, the tumor cells may remain dependent on androgen signaling, which has led to the development of androgen deprivation therapies. However, in some patients, the disease eventually progresses to castration-resistant prostate cancer (CRPC) and ultimately evolves into metastatic castration-resistant prostate cancer (mCRPC) [8].

A crucial step in the progression of PCa is the epithelial–mesenchymal transition (EMT) [9,10]. During this process, tumor cells lose epithelial characteristics and acquire a mesenchymal phenotype that makes them more mobile and invasive, favoring dissemination, especially towards bones, the most common metastatic site.

The tumor microenvironment (TME) is a dynamic and complex ecosystem that plays an essential role in the initiation, progression, and therapeutic resistance of PCa [11,12]. The stromal compartment, including cancer-associated fibroblasts (CAFs) [13], releases transforming growth factor-β (TGF-β), interleukin-6 (IL-6), and vascular endothelial growth factor (VEGF) [14]. These molecules remodel the extracellular matrix (ECM) and drive angiogenesis, promoting tumor growth and creating barriers to drug delivery. The TME is characterized by a paucity of tumor-infiltrating lymphocytes (TILs) and an abundance of immunosuppressive cells, including regulatory T cells (Tregs), myeloid-derived suppressor cells (MDSCs), and M2-polarized tumor-associated macrophages (TAMs). These components collectively dampen cytotoxic CD8⁺ T-cell activity, thereby enabling tumor cells to evade immune-mediated destruction [15].

Chronic inflammation contribute to the onset and progression of PCa, by remodeling the ECM, triggering EMT, and releasing cytokines and growth factors that boost cell proliferation and facilitate tissue invasion [16]. Furthermore, hypoxia in the PCa microenvironment stimulates angiogenesis, alters metabolism, and remodels the ECM, driving cancer cells toward a more aggressive and metastatic phenotype [17]. The interaction between stromal and immune components establishes a feedback loop that sustains tumor expansion and resistance to therapy. Signals from CAFs attract and polarize immune cells toward suppressive phenotypes, while hypoxia enhances pro-tumor cytokine secretion and supports CRPC evolution [18].

One of the most studied aspects in PCa concerns the genetic and epigenetic alterations that characterize the pathogenesis. The most frequent chromosomal abnormality is a fusion between the oncogenic transcription factor ERG (EST-related gene) and TMPRSS2 (5′-transmembrane protein serine proteinase-2) which drives carcinogenesis in over 50% of patients with PCa [1,19]. Other alterations frequently observed in aggressive forms include the loss or mutation of tumor suppressor genes [20], such as BRCA1/BRCA2 (breast cancer), PTEN (phosphatase and tensin homolog), p53 and RB (retinoblastoma), and the activation of PI3K/Akt pathway that promote proliferation and reduce sensitivity to apoptotic stimuli. DNA methylation and other epigenetic modifications can silence genes that control cell growth, contributing to malignant transformation [21].

PCa treatment strategy depends on tumor stage, risk category, PSA levels, Gleason score, and overall health [1,22]. For low-risk, slow-growing tumors, active surveillance is a viable strategy. When treatment is required, radical prostatectomy, often robot-assisted, and various forms of radiation therapy, including external beam radiotherapy and brachytherapy, are standard curative approaches [23]. In advanced or metastatic cases, hormone therapy is essential [24]. Androgen deprivation therapy (ADT) with GnRH agonists or antagonists reduces testosterone levels that fuel PCa growth [25]. When hormone therapy becomes insufficient, second-generation anti-androgens, such as enzalutamide, or androgen synthesis inhibitors, like abiraterone, are used to further suppress progression [26]. Chemotherapy with docetaxel or cabazitaxel is standard once resistance to abiraterone or enzalutamide develops [27].

**Figure 1:**
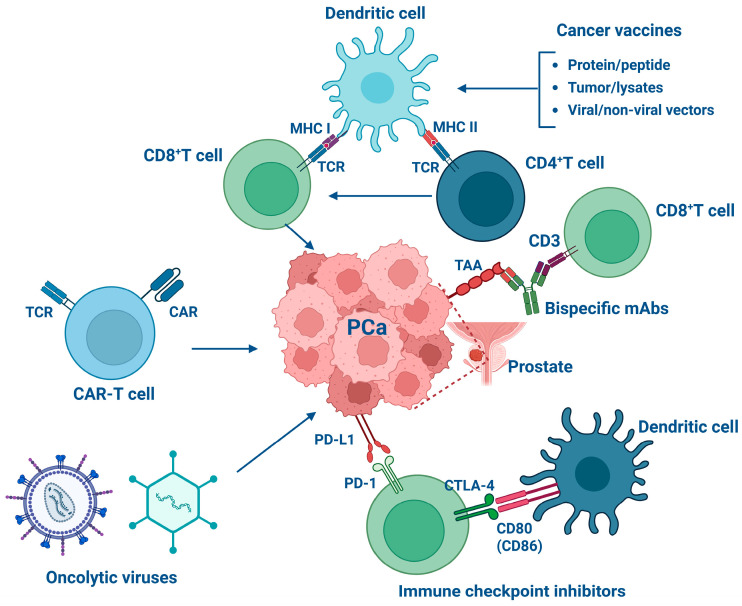
Overview of PCa immunotherapy. Created in BioRender. Zappalà, M. (2025) https://BioRender.com/bdivqnb.

Immunotherapy represents an innovative frontier in the treatment of hematological malignancies and certain solid tumors [28,29,30]. However, its application in PCa faces challenges due to the highly immunosuppressive TME and the low intrinsic immunogenicity of malignant prostatic cells [27,31,32]. As a result, PCa is considered an immune-resistant “cold” tumor in which immunotherapeutic strategies often yield limited success [33]. Moreover, identifying tumor-specific antigens is difficult because malignant and normal prostatic tissues share many molecular features, reducing immune visibility. Nevertheless, modulation of immune pathways can restore antitumor reactivity even in such suppressive environments, enabling activation and expansion of tumor-specific T cells. Current research focuses on immune checkpoint inhibitors (ICIs), bispecific antibodies (bsAbs), cancer vaccines, and chimeric antigen receptor T (CAR-T) cells, approaches that may together enhance the effectiveness of immunotherapy in PCa (Figure 1).

In this review, after briefly outlining the principal immunotherapeutic approaches, we focus on recent advances in CAR-T cell therapy for prostate cancer. We discuss the molecular targets, preclinical and clinical developments, and the main biological and translational obstacles that limit its efficacy, as well as the strategies designed to overcome them.

## 2. Current Immunotherapeutic Approaches for PCa

Immunotherapeutic approaches use the immune system’s responses to fight tumor growth. From the first applications [34,35], cancer immunotherapy has shown promising potential, but a more limited efficacy, compared to other malignancies, in the treatment of cold tumors, especially PCa [27,36]. The limited effectiveness of immunotherapy in PCa reflects both tumor-intrinsic properties and a profoundly suppressive TME [11]. Furthermore, despite expressing several tumor-associated antigens (TAAs), PCa remains poorly immunogenic largely due to a low mutation rate and efficient DNA-repair mechanisms. This results in limited neoantigen generation and diminished antigen presentation, which in turn reduces immune cell infiltration and promotes immune evasion [27]. Collectively, these factors create a hostile microenvironment that restricts antitumor immunity and represents a major obstacle to effective immunotherapeutic design in PCa [37,38].

### 2.1. Immune Checkpoint Inhibitors

Immune checkpoints are immune-regulatory pathways that help maintain self-tolerance, prevent autoimmunity, and reduce collateral tissue damages [39]. Cancer cells exploit these pathways to suppress immune surveillance, upregulating inhibitory ligands that dampen T-cell effector functions and allow uncontrolled proliferation [40]. This discovery has highlighted the critical role of T lymphocytes in controlling tumor growth and has led to a new therapeutic approach using ICIs.

ICIs are monoclonal antibodies (mAbs) that block the interaction between inhibitory receptors on T cells and their ligands, thereby preventing the suppression of antitumor immune responses [41]. By interrupting the binding between receptors and tumor-derived ligands, such as cytotoxic T lymphocyte-associated protein 4 (CTLA-4), programmed cell-death protein-1 (PD-1) and programmed death-ligand-1 (PD-L1), these mAbs have revolutionized the treatment of several malignancies. However, in prostate cancer, the benefits of ICIs have been modest. Monotherapy with ICIs does not sufficiently overcome the cold TME and the immune resistance mechanisms characteristic of PCa [42,43].

In a phase III clinical trial, the human anti-CTLA-4 mAb ipilimumab demonstrated only limited benefits in patients with mCRPC. Although some patients experienced prolonged progression-free survival and increased immune cell infiltration in the typically “cold” tumor, no statistically significant improvements in overall survival were observed compared with placebo [44,45,46].

Efficacy is similarly limited with anti-PD-1/PD-L1 mAbs. Pembrolizumab produced durable objective responses in only a subset of heavily pretreated, PD-L1-positive PCa patients, despite maintaining a favorable safety profile (NCT02054806) [47]. After four years of follow-up, pembrolizumab monotherapy continued to show modest activity and manageable toxicity in docetaxel-pretreated mCRPC patients (NCT02787005) [48,49].

Most prostate cancers have a low proportion of tumor-infiltrating lymphocytes and low expression levels of TAAs and neoantigens, making them immune-resistant cold tumors. Only a minority of patients benefit from ICI therapy. Furthermore, the enrichment of immunosuppressive cells, cytokines, and chemokines promotes T-cell exhaustion and hinders trafficking into tumor sites [50].

Recognizing these limitations of ICI monotherapy, numerous clinical trials are now investigating combination strategies to improve immune efficacy. These include pairing ICIs with other checkpoint inhibitors or integrating them with therapies such as radio/chemotherapy, or hormonal therapy. For example, combining ipilimumab with nivolumab, a human anti-PD-1 mAb, produced measurable antitumor activity in both chemotherapy-naïve and chemotherapy-experienced mCRPC patients. However, these findings are tempered by the small patient cohort and short follow-up duration [51]. Improved response to ICIs has been observed in cancer patients carrying specific molecular alterations such as high tumor mutational burden (TMB), microsatellite instability-high (MSI-H) or mismatch repair deficiency (dMMR). In a clinical study (NCT02985957), only 4 of 90 mCRPC patients with relatively high TMB achieved durable clinical responses and complete tumor regression when treated with nivolumab plus ipilimumab, before or after chemotherapy [51].

Similarly, a subset of patients with MSI-H or dMMR disease experienced PSA decline and prolonged survival after pembrolizumab, reinforcing the therapeutic potential of anti-PD-1/PD-L1 antibodies in biomarker-defined populations [52].

In addition, many clinical trials suggest that the use of ICIs may be an effective and safe strategy in optimizing CAR-T cell therapy and may improve the efficacy and persistence of CAR-T cells in cancer patients, as described in the next section [53].

### 2.2. Bispecific Antibodies

Bispecific antibodies (bsAbs) represent a promising immunotherapeutic approach for advanced PCa [54]. These engineered molecules can simultaneously bind two distinct antigens, typically one expressed on tumor cells, e.g., Prostate-Specific Membrane Antigen (PSMA), and another on immune effector cells, such as CD3 or CD28 on T cells, thereby promoting an immunological synapse that enables targeted immune activation [55,56]. The modular structure of bsAbs provides substantial design flexibility, allowing for the targeting of alternative antigens or the incorporation of additional functional domains to enhance specificity and minimize on-target off-tumor effects. This versatility may help overcome tumor immune escape mechanisms associated with low neoantigen presentation and an immunosuppressive microenvironment. Furthermore, optimized bsAb design may optimize dosing regimens and administration protocols to maximize efficacy while reducing adverse effects, particularly cytokine release syndrome (CRS) [54,57].

Among the most clinically advanced bsAbs are pasotuxizumab, acapatamab and CC-1, all bispecific PSMAxCD3 antibodies that differ in their molecular architecture [36,54]. Pasotuxizumab consists of two single-chain variable fragments (scFvs) fused together; acapatamab links two scFv fragments to an IgG single-chain Fc region that interacts with the neonatal Fc receptor (FcRn), whereas CC-1 is designed as an IgG-based construct in which the scFv fragments are covalently linked to the Fc region of an IgG1 antibody. These structural differences influence the half-life and consequently the clinical administration strategies: pasotuxizumab typically requires continuous infusion due to its smaller size and rapid clearance [58], whereas acapatamab and CC-1 have been engineered for improved pharmacokinetics, potentially allowing for intermittent dosing [59]. Furthermore, these differences may influence their binding affinities, safety profiles, and overall clinical efficacy, though direct comparative studies are still needed to fully establish these distinctions.

Preliminary findings from early-phase clinical trials evidenced that pasotuxizumab has an acceptable safety profile with manageable side effects in patients with advanced PCa. These studies have also demonstrated early signs of antitumor activity, including reductions in PSA levels and evidence of tumor shrinkage in some patients. However, further investigations are required to confirm its therapeutic efficacy and establish optimal dosing parameters [58]. Regarding acapatamab, its interaction with FcRn has been shown to significantly extend its half-life [60]. Despite this pharmacokinetic advantage, the manufacturer, Amgen, has decided to discontinue clinical development of acapatamab [61].

CC-1 is a promising bispecific antibody with a favorable safety profile and encouraging clinical activity, showing a rapid and substantial reduction in PSA levels in most treated patients [59].

Several novel bsAbs constructs are currently under development targeting alternative PCa antigens, including glypican-1 [62], a disintegrin and metalloproteinase 17 (ADAM17), also known as TNF-α converting enzyme (TACE) [63], prostate stem cell antigen (PSCA) [64], human kallikrein 2 (KLK2) [65], and six-transmembrane epithelial antigen of prostate 1 (STEAP1) [66].

Among the most promising candidates is JNJ-78278343, a bsAb designed to targets KLK2 on tumor cells and the CD3 receptor complex on T cells. Phase I clinical trials are ongoing to determine the recommended phase II doses (RP2Ds) and assess the safety of JNJ-78278343, both as monotherapy (NCT04898634) or in combination with another bsAb, JNJ-87189401, which targets PSMA and CD28 (NCT06095089).

Another promising candidate is xaluritamig (AMG 509), a bsAb that targets STEAP1 and CD3. Clinical studies have demonstrated that bsAbs targeting STEAP1 can be effective treatments for advanced PCa, with xaluritamig producing significant radiographic and PSA responses, confirming STEAP1 as a viable cancer target [66]. Ongoing research is focused on determining optimal dosing and evaluating combinations with standard treatments, such as abiraterone or enzalutamide, paving the way for future trials in advanced PCa management (NCT04221542).

Despite their therapeutic potential, bsAb therapies face challenges, including short half-life, high risk of CRS, and potential treatment resistance. Ongoing clinical trials are focused on optimizing dosing schedules and administration strategies to improve bsAb therapeutic outcomes. Combination approaches with other immunomodulatory agents, such as ICIs, are under investigation. By simultaneously blocking inhibitory pathways and promoting direct T cell engagement, these combination strategies may synergistically improve clinical outcomes in patients with advanced PCa [67,68].

### 2.3. Cancer Vaccines

Cancer vaccines represent another targeted therapeutic strategy designed to elicit robust potent T cell responses against TAAs [69].

The dendritic vaccine sipuleucel-T remains the only FDA-approved immunotherapy for PCa and the first therapeutic vaccine approved for any cancer [70]. Sipuleucel-T is an autologous cellular immunotherapy designed to induce a targeted immune response against prostatic acid phosphatase (PAP), an antigen expressed in most prostate cancers [71]. Peripheral blood mononuclear cells are collected from the patient and cultured with PAP-GM-CSF, a fusion protein consisting of PAP linked to granulocyte macrophage colony-stimulating factor (GM-CSF), an immune cell activator [72]. During ex vivo culture, activated antigen-presenting cells (APCs) internalize and process the recombinant antigen, presenting PAP-derived peptides to T cells [73]. After the incubation, the activated cells are reinfused into the patient.

The phase III IMPACT trial (NCT00065442) demonstrated a four-month increase in overall survival in patients with metastatic PCa compared with placebo [74]. Sipuleucel-T treatment is generally well tolerated, with the most common adverse events being chills, fatigue, pain and low-grade fever [74,75,76]. The FDA approved Sipuleucel-T in 2010 for the treatment of asymptomatic or minimally symptomatic mCRPC [77]. The vaccine was also approved by the European Medicines Agency (EMA) in 2013; however, in 2015, the manufacturer Dendreon requested its withdrawal for commercial reasons and not because of safety or efficacy concerns [78].

Beyond dendritic cell vaccines, several novel cancer vaccine platforms have been developed, including peptide-based, viral vector and DNA/RNA vaccines [79].

Peptide vaccines employ short protein fragments derived from tumor-associated antigens to induce robust and specific immune responses. Promising results have been reported with a vaccine targeting the Ras homolog gene family member C (RhoC), a small GTPase overexpressed in advanced solid cancers, metastases and cancer stem cells [80]. In post-prostatectomy patients, RhoC-directed vaccination elicited a sustained T-cell response and demonstrated a favorable safety and tolerability profile (NCT03199872) [81].

Viral vector vaccines use genetically modified viruses to deliver tumor-associated genetic material, thereby inducing recognition and cytotoxic responses against cancer cells. PROSTVAC is composed of a recombinant vaccinia vector containing transgenes for human PSA and three costimulatory molecules: B7.1 (CD80), leukocyte function-associated antigen-3 (LFA-3, CD58), and intercellular adhesion molecule-1 (ICAM-1, CD54) [82]. This construct is designed to enhance T-cell activation and promote the lysis of tumor cells [83]. Although phase II trials reported encouraging results, including improved median overall survival [84], the subsequent phase III study (NCT01322490) failed to demonstrate a statistically significant survival benefit compared with placebo [85].

Nucleic acid vaccines use genetic material derived from cancer cells to stimulate the immune system. In PCa, most DNA vaccine candidates target specific antigens, such as PAP or PSA or androgen receptors. For instance, the most advanced candidate, pTVG-HP, uses a plasmid to express human PAP to stimulate antigen-specific T-cell responses. However, results from a phase II clinical trial indicated that pTVG-HP did not lead to a statistically significant improvement in two-year metastasis-free survival (NCT01341652) [86].

### 2.4. Oncolytic Viruses

Oncolytic virotherapy is an emerging anticancer strategy that employs genetically engineered viruses to selectively replicate in and lyse cancer cells, while sparing healthy tissue [87,88]. Building on advances in immunotherapy, oncolytic adenovirus-based treatments are being actively explored as a promising approach to improve therapeutic outcomes in PCa [89,90]. Both preclinical and clinical studies have confirmed the antitumor potential and safety of oncolytic adenoviruses in PCa [91,92,93].

Oncolytic viruses not only induce direct tumor lysis but also transform the immunologically ‘cold’ prostate tumor microenvironment into a ‘hot’ one by releasing tumor antigens, proinflammatory cytokines, and danger-associated molecular patterns (DAMPs). These effects recruit and activate dendritic cells and cytotoxic T lymphocytes. Importantly, oncolytic virotherapy can be strategically combined with CAR-T cells, serving as an in situ vaccine that enhances T-cell trafficking and persistence, thereby amplifying the overall antitumor immune response [94].

A phase I trial evaluating oncolytic adenovirus-mediated cytotoxic and IL-12 gene therapy for locally recurrent PCa after definitive radiotherapy demonstrated that the replication-competent Ad5-IL-12 adenovirus (Ad5-yCD/mutTKSR39rep-hIL-12) was well tolerated when delivered directly to prostate tumors (NCT02555397) [95].

Similarly, a phase I/IIa first-in-human study evaluating the intratumoral administration of ORCA-010 in treatment-naïve PCa patients showed an excellent safety profile, with no dose-limiting toxicities and only transient mild adverse events. Preliminary results indicate successful viral replication, early signs of antitumor activity, and a reduction in prostate size among patients with prostate enlargement (NCT04097002). The increase in tumor-infiltrating and circulating prostate-specific T cells, along with stable regulatory T cells, indicates a favorable immune environment [96].

Despite promising preliminary results, several significant challenges remain. Key limitations include viral immunogenicity, tissue-specific targeting, and complex virus–host interactions that are not yet fully understood. Optimization strategies such as genetic modification, cytokine payloads, and carrier systems are being explored to improve efficacy. Nonetheless, most oncolytic adenoviruses remain in early-stage trials, and pre-existing immunity in humans may ultimately limit their therapeutic potential [87,88].

## 3. Chimeric Antigen Receptor T-Cells

Chimeric Antigen Receptor T (CAR-T) cells therapy represents a pivotal branch of tumor immunotherapy [97]. This approach is aimed at recognizing and specifically eliminating target cells that express surface antigen epitopes. CAR-T therapy has achieved remarkable clinical success of hematological malignancies [98,99] and holds significant promise as a therapeutic strategy for solid tumors [100,101], including prostate cancer [102].

CAR-T therapy employs T cells genetically engineered to express chimeric receptors that combine the target specificity of monoclonal antibodies with the cytotoxic power of T cells. This innovative design enables them to precisely identify and eliminate tumor cells. Typically, T cells are collected from the patient, genetically modified to enhance their antitumor activity, expanded in vitro and subsequently reinfused into the patient [103]. The efficacy of CARs depends on their modular structure that integrates three different functional domains, ectodomain, transmembrane domain and endodomain, each with a well-defined role in signal transduction and T cell activation. Optimizing these components is essential to improve efficacy and reduce adverse effects.

The ectodomain is responsible for antigen recognition and binding. It typically contains an antigen-binding domain (ABD) and a hinge/spacer region. The ABD usually consists of variable heavy (VH) and light (VL) chains from mAbs joined by a linker to form a single-chain variable fragment (scFv). This scFv ensures high affinity and specificity in the MHC-independent recognition of antigens expressed on target cell surfaces, thereby directing the cytotoxic activity of T cells. Selective antigen recognition is crucial to avoid off-target activation that could damage healthy cells. However, scFvs may induce self-aggregation and immunogenicity, leading to premature T-cell exhaustion [104]. Furthermore, drawbacks include limited targeting of intracellular antigens and the risk of treatment failure due to antigen loss or downregulation [105]. Alternative recognition domains, such as camelid-derived nanobodies (V_H_Hs), can access epitopes that are sterically hindered in solid tumors [106,107].

Immediately following the recognition domain is the hinge domain (HD), also referred to as the spacer region. This portion provides the necessary flexibility for the scFv domain to effectively interact with the antigen, even in the presence of variations in the distance or conformation between the T cell and the target cell. Longer HDs enhance flexibility, allowing access to membrane-proximal epitopes or complex glycosylated antigens, while shorter hinges are better suited for targeting membrane-distal epitopes [108,109]. HDs often derive from constitutive regions of immunoglobulins (such as IgG) and might trigger innate immune responses, because of the ability of CH2 region to bind to Fcγ receptors expressed by innate immune cells [110]. Modifying or removing the CH2 domain decreases immune responses and restores CAR T cell activity [111].

The transmembrane domain (TMD) connects the extracellular and intracellular parts of the CAR [112]. TMD derives from different natural proteins, including CD3ζ, CD4, CD8α, or CD28. The choice of TMD influences the stability of the receptor on the membrane and its ability to interact with endogenous signaling molecules [112,113]. Actually, CD28 is considered the more stable TMD [114].

The endodomain of a CAR-T cell, also known as intracellular signaling domain (ISD), is essential to translate antigen recognition signals, activate the T cell and trigger an immune response [111]. ISD typically contains a primary activation domain and one or multiple costimulatory domains. The primary activation domain is the CD3ζ protein, naturally found in the T-cell receptor (TCR) complex [115]. CD3ζ contains immunoreceptor tyrosine-based activation motifs (ITAMs), which are essential for the initial activation of T cells through phosphorylation by protein kinases. This cascade promotes tumor cytolysis and cytokine secretion [116].

**Figure 2:**
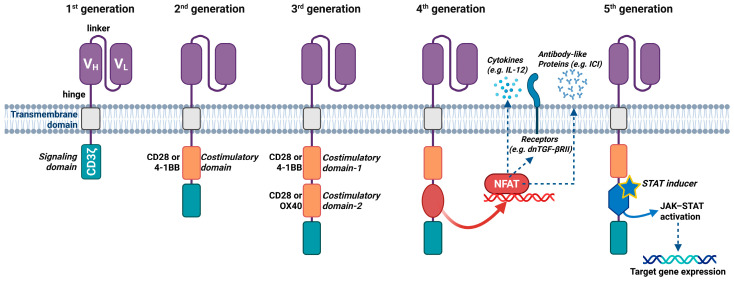
CAR generation constructs with the progressive modifications to their intracellular signaling domains. Created in BioRender. Zappalà, M. (2025) https://BioRender.com/nv4axys.

In second-, third-, and later-generation CARs, the CD3ζ domain is accompanied by one or more costimulatory domains (e.g., CD28, 4-1BB, OX40) [117]. These domains enhance T-cell activation, proliferation and persistence, contributing to a more robust and longer-lasting immune response. Based on the organization of the endodomain [118], five generations of CAR-T cells have been developed, each improving in efficacy and safety compared to the previous one (Figure 2).

First-generation CAR-T cells were engineered by combining an scFv fragment with a single intracellular CD3ζ signaling domain. Although this domain can trigger ITAM phosphorylation and elicit an effective cytotoxic response, it is insufficient for complete tumor cell eradication. The lack of costimulatory domains resulted in a limited response in terms of persistence, proliferation and cytokine secretion. This ultimately led to rapid T cell exhaustion and reduced clinical efficacy, particularly in the immunosuppressive microenvironment of solid tumors [112,119].

To overcome these limitations, second-generation constructs integrated an additional costimulatory domain alongside CD3ζ [120]. This integration significantly improved cytokine secretion, proliferation and survival of CAR-T cells, leading to a more durable immune response and greater tumor clearance [117]. The costimulatory domains are derived from two receptor family, the CD28 receptor family, e.g., CD28 and inducible T cell costimulator (ICOS), and the tumor necrosis factor (TNF) receptor family, such as 4-1BB (CD137), OX40 (CD134) and CD27 [121,122]. Costimulation via CD28 is crucial for enhancing clonal proliferation, survival and differentiation into effector and memory T cells [123,124]. It also boosts cytokine production, especially IL-2, a key growth factor for T cell. ICOS has similar effects but induces higher levels of IL-10, a B-cell differentiation factor [125]. OX40 promotes sustained T cell proliferation and robust IL-2 production, while 4-1BB favors memory CD8+ T cells generation and enhances CAR T cell persistence [126,127]. Similarly, CAR constructs with CD27 demonstrate enhanced persistence and resistance to apoptosis [117].

Third-generation CAR-T cells further enhanced T-cell activation by incorporating two costimulatory domains, such as CD28 and 4-1BB or CD28 and OX40 [117]. This dual signaling improved T-cell persistence, proliferation, and cytokine production. As a result, these cells displayed faster and stronger cytotoxic activity and were more effective against resistant tumors [128,129,130].

Fourth-generation CAR constructs were specifically designed to counteract the immunosuppressive microenvironment of solid tumors. Their intracellular signaling domain is derived from 2nd-generation CARs but includes additional functional elements [131]. Often referred to as armored CAR-T cells, these constructs are engineered to express transgenic proteins, typically cytokines that may be produced either inducibly or constitutively [132].

Armored CAR-T cells can be categorized into three types: T cells redirected for universal cytokine killing (TRUCK), cytokine-modulating CAR-T cells and antibody-secreting CAR-T cells [133]. TRUCK CAR-T cells extend 2nd-generation designs by incorporating inducible transgene cassettes controlled by a nuclear factor of activated T cells (NFAT) responsive promoter. These cassettes drive cytokine expression (e.g., IL-12, IL-15, IL-7, or IL-21) only upon antigen engagement [131,134,135]. The local release of proinflammatory cytokines helps reprogram the TME, making it a less hostile environment for immune activity and enhancing antitumor efficacy.

Beyond secreting proinflammatory cytokines, CAR-T cells can also be reprogrammed to modulate their cytokine responsiveness. This is achieved by equipping them with decoy or switch cytokine receptors. These cytokine-modulating CAR-T cells express tailored extracellular receptor domains that sequester inhibitory cytokines (e.g., TGF-β, IL-4) at the cell surface, thereby blocking downstream immunosuppressive signaling in the TME [131,136]. For example, PSMA-targeting CAR-T cells co-expressing a dominant-negative TGF-β receptor II (dnTGF-βRII) can block TGF-β signaling. This modification reduces T cells exhaustion, enhances intratumoral proliferation, and improves safety in preclinical prostate-cancer models [137].

Antibody-secreting CAR-T cells combine the tumor-targeting precision of CAR-T therapy with the localized delivery of therapeutic antibodies. This dual approach strengthens antitumor effects while reducing systemic toxicity [138]. These CAR-T cells are engineered to coexpress secretion cassettes encoding scFvs against immune checkpoints, particularly PD-1. Upon antigen engagement, PD-1 scFvs are released directly into the TME, where they block PD-1/PD-L1 interactions and reverse local immunosuppression [139,140]. This bifunctional approach amplifies antitumor efficacy and T-cell persistence while minimizing systemic immune-related toxicities. Early clinical studies in ovarian cancer demonstrate promising responses with reduced off-tumor effects [141]. Overall, 4th-generation CAR-T cells represent a significant advance in treating solid tumors, which traditionally respond poorly to CAR-T therapy. They also offer a safer approach by avoiding the continuous, high-level expression of potent cytokines [142].

Fifth-generation CARs retain the basic architecture of 2nd-generation constructs, including the scFv, hinge, transmembrane region, costimulatory domain, and CD3ζ. However, they include an additional truncated IL-2Rβ cytoplasmic fragment containing STAT3/5 recruitment motifs. This fragment is inserted between the costimulatory domain (e.g., CD28 or 4-1BB) and CD3ζ, enabling antigen-triggered JAK–STAT signaling [143,144,145]. Upon antigen engagement, 5th-generation CAR-T cells deliver three synergistic signals, (1) TCR-like activation via CD3ζ ITAMs, (2) co-stimulation via CD28 (or 4-1BB), and (3) JAK–STAT signaling through IL-2Rβ. Together, these signals mimic physiological T-cell priming, promoting stronger expansion, enhanced cytokine secretion, and the development of central memory phenotypes [145,146]. In preclinical models, 5th-generation CAR-T cells have shown improved tumor clearance, greater persistence, and lower expression of exhaustion markers compared to 2nd-generation CARs [144,147].

The progressive development of CAR-T cell generations has been driven by the goal of markedly improving T cell activation. However, these advancements have also led to more pronounced adverse effects, primarily due to excessive immune activation and on-target off-tumor toxicity.

**Figure 3:**
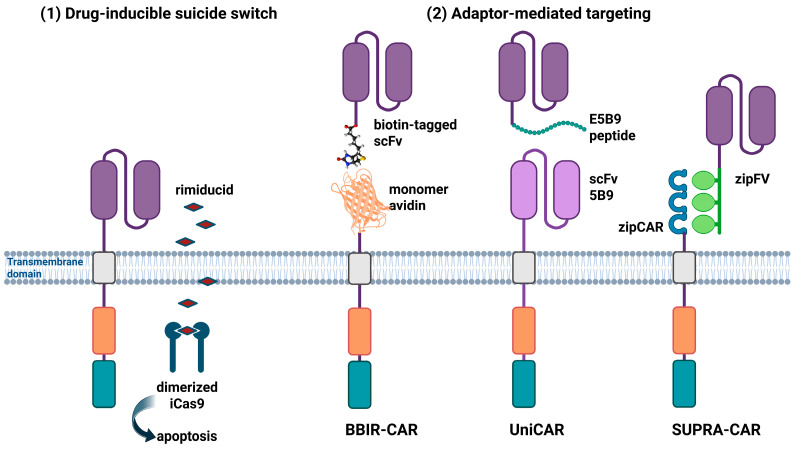
Drug-inducible suicide switch (1) and adaptor-mediated targeting (2) approaches. Created in BioRender. Zappalà, M. (2025) https://BioRender.com/t6sxxzi.

To improve the safety of CAR-T cell therapy without compromising efficacy, regulatory modules can be incorporated to allow controlled activation. This control can be achieved through genetic circuit integration or modification of the T cell chassis (Figure 3) [148]. One of the most effective and straightforward strategies for mitigating on-target off-tumor effects is the use of inducible suicide switches, such as the iCas9 system [149]. The iCas9 safety switch includes a caspase-9 domain fused to a drug-binding domain from the mutant hFK506-binding protein (FKBP12-F36V). This construct is linked to the CAR gene via a short 2A “self-cleaving” peptide (T2A element). When a chemical inducer of dimerization (CID), such as rimiducid, is administered, it activates caspase-9. This activation initiates an apoptotic cascade that selectively eliminates CAR-T cells, thereby improving their safety profile [114,150].

Other strategies to overcome the limitations of CAR-T therapy in solid tumors focus on a novel class of modular, engineered CAR-T cells. These cells require co-administration of an adapter molecule that connects the CAR-T cell to its target, activating its tumor-killing function.

Adapter CARs are engineered by replacing the traditional cancer-specific scFv extracellular domain with an inert recognition domain. This domain binds a secondary adapter molecule that targets the cancer-specific antigen (Figure 3) [151]. By modulating the concentration and timing of adapter administration, the therapeutic effect can be precisely tuned or rapidly halted. This control helps prevent or reduce CRS-like toxicity [152].

Adapter switches also enable quick retargeting toward different tumor antigens, enhancing both flexibility and cost-efficiency. This modular strategy provides greater versatility than conventional CAR-T cells, enabling them to target a broader range of cell types with improved precision [153].

The Biotin-Binding Immune Receptor CAR (BBIR-CAR) is a modular CAR design in which the T cell expresses an extracellular monomeric avidin (streptavidin) domain linked to intracellular signaling regions [153]. This system redirects T cell cytotoxicity through cancer-targeted, biotinylated antibody adapter molecules [154]. BBIR-CAR T cells remain inactive until a biotinylated adapter is supplied. When a biotin-tagged antibody binds to a tumor antigen, the avidin domain recognizes the biotin, triggering T-cell activation and cytotoxicity [155]. Due to the inherent modularity of the avidin scaffold, a single BBIR-CAR T-cell product can be reprogrammed to target different antigens simply by providing distinct biotinylated antibodies. This flexibility allows simultaneous or sequential targeting of multiple tumor-associated antigens [156].

T-cell activation strictly depends on the presence of the adapter molecule. This property enables precise temporal and dose control, enhancing safety. Importantly, physiological biotin levels do not significantly activate or inhibit BBIR-CARs, minimizing off-target effects [153].

The UniCAR system is another switchable and universal CAR platform. It employs a two-component design in which each element is inactive on its own but activates T cells when combined [153]. The system includes (1) an inert scFv (5B9) CAR extracellular domain that specifically binds a short peptide antigen (E5B9), and (2) the E5B9-tagged targeting molecule (TM) that binds to tumor to antigens on cancer cell surfaces [157]. When these two components interact, the scFv binds to the E5B9 peptide on the TM, forming a functional bispecific immune complex that triggers T cell cytotoxic response [158]. UniCAR provides precise temporal control of T cell activity, directing tumor cell killing through recognition of E5B9-tagged therapeutic antibody TMs [159]. Any TM can be exchanged to redirect the same CAR T cells toward new antigens, even multiple antigens, simultaneously or sequentially.

This adaptability reduces the risk of antigen-loss relapse [157]. UniCAR T cells can also be repeatedly activated by infusing the TM and deactivated simply by halting it, as the TM is quickly cleared from the body. This fast clearance is essential for safely managing potential side effects [160].

The split, universal, and programmable SUPRA CAR platform comprises two modular components. The first is a universal receptor, called zipCAR, expressed on engineered T cells, which combines a leucine zipper motif with an intracellular signaling domain. The second is a separate switch molecule (SM) that carries a matching leucine zipper fused to a tumor-targeting scFv fragment (zipFv) [161].

When the complementary zippers dimerize, the extracellular signal is transmitted to the intracellular domain, triggering T cell activation [161]. By introducing different zipFv proteins, a T cell expressing the universal zipCAR can be redirected toward various tumor antigens [162].

A key advantage of this system is its reversible control. Adding a higher-affinity zipFv can replace the original one, effectively turning the switch off [163]. Furthermore, zipFv variants with different leucine zipper affinities can fine-tune the intensity of T-cell activation through competitive binding. This adjustability enhances both the safety and efficacy of T-cell therapy [164].

The BBIR-CAR, UniCAR, and SUPRA-CAR technologies were all designed as modular and flexible platforms for use with both autologous and allogeneic T cells. Although their modular nature opens the possibility for future “off-the-shelf” use of allogeneic T cells, most preclinical and early clinical studies have so far employed autologous T cells due to safety and immune compatibility considerations.

### 3.1. Production of CAR-T Cells

**Figure 4:**
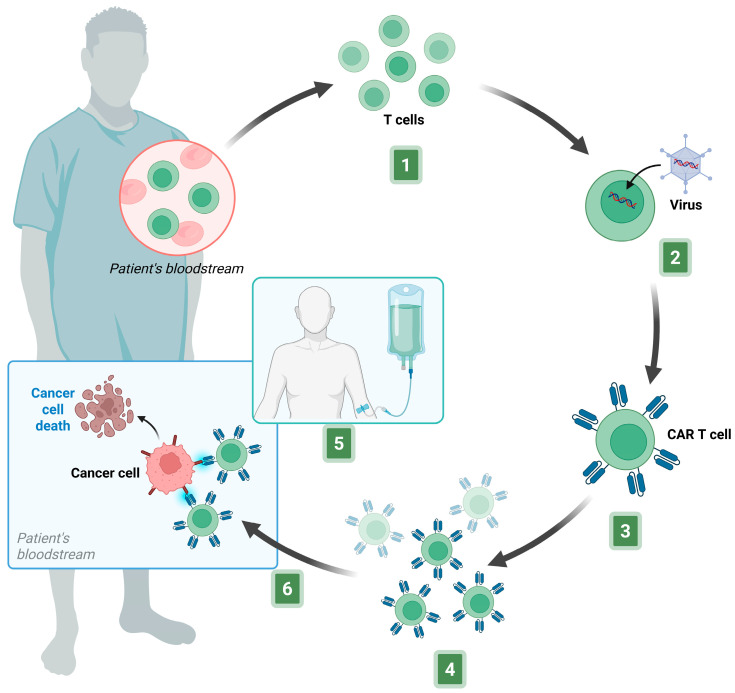
Main stages of autologous CAR-T cell production. (1) T cell collection and activation. (2) Insertion of CAR transgene into the T cells. (3) CAR-T cell generation. (4) CAR-T cell expansion. (5) Lymphodepleting chemotherapy. (6) CAR-T cells infusion. Created in BioRender. Zappalà, M. (2025) https://BioRender.com/ctq17my.

The production of CAR-T cells is a highly sophisticated biotechnological process that combines advanced genetic engineering, cell culture techniques, and rigorous quality control procedures. All steps are performed in compliance with Good Manufacturing Practices (GMPs) standards. This process is time-consuming and expensive, usually requiring 2–4 weeks to complete before the cells can be administered to the patient. It includes several key steps, each of which is essential to ensuring the safety and efficacy of the final therapeutic product (Figure 4).

CAR-T cells can be generated from the patient’s own T cells (autologous) or from those of a healthy donor (allogeneic). The CAR-T cell products currently on the market are autologous. However, to improve treatment accessibility, researchers are exploring universal CAR-T cell therapies based on donor-derived T cells [165].

The manufacturing process typically starts with a leukapheresis procedure to collect the patient’s peripheral blood mononuclear cells. These cells are enriched for T-cells through negative selection to remove unwanted cell types. Separation is usually performed using flow cytometry, which sorts cell by size, or magnetic bead-based methods that exploit specific markers expressed on the cell membrane. These techniques enrich the T lymphocytes population essential for subsequent genetic engineering [97,166,167]. The isolated T cells are then activated ex vivo through stimulation with specific antibodies, commonly anti-CD3 and anti-CD28, which mimic natural activation and proliferation signals. This step is crucial to prepare the cells for genetic modification and to enhance transduction efficiency. CAR gene transduction into autologous T lymphocytes is typically achieved using viral vectors, most often lentiviruses or retroviruses. These vectors are designed to ensure stable and long-lasting integration of the transgene, allowing sustained CAR expression. Despite their efficiency, viral vectors present manufacturing and biosafety limitations, including high production costs, long lead times, and the potential for insertional mutagenesis. For this reason, in recent years, alternative methods based on gene-editing technologies such as Clustered Regularly Interspaced Short Palindromic Repeats/Caspase 9 (CRISPR/Cas9), have also been explored to have the possibility of introducing targeted modifications and reducing the risk of unwanted insertion events. Other non-viral delivery systems, such as transposon-based technologies (Sleeping Beauty, PiggyBac) or mRNA transfection, enable transient or site-specific CAR expression, improving safety while maintaining antitumor activity [129,168].

The subsequent CAR-T cells expansion step is realized in vitro using bioreactors or controlled cell cultures. The culture medium is enriched with cytokines, such as IL-2, IL-7, IL-15, IL-21, and TGF-β1, which promote proliferation, differentiation, and survival of the engineered T-cells. During this phase, critical parameters including cell density, phenotype and CAR expression must be continuously monitored to ensure the cells retain the desired functional characteristics. Advanced automated closed-system bioreactors have recently been developed to streamline this process under sterile, monitored conditions, reducing operator variability and contamination risks while ensuring product consistency.

Finally, quality control tests are performed to verify purity and viability of the CAR-T cells, correct transgene integration and expression, and cytotoxic activity against target tumor cells: These tests also ensure the absence of microbial contamination or residual viral vectors. In some cases, molecular assays are conducted to monitor transgene integration and exclude undesirable mutations or insertional events that could compromise patient safety. Once all quality criteria are met, CAR-T cells are formulated as the final product and cryopreserved until infusion. Before administration, patients undergo lymphodepletion with standard chemotherapy (cyclophosphamide and fludarabine) to create a favorable environment for CAR-T cell expansion and antitumor activity [166,169].

### 3.2. Prostate Tumor Antigens as Target for CAR-T Therapy

An ideal target for CAR-based therapy in PCa must meet several critical criteria. First, it should be robustly and uniformly expressed on PCa cells, with minimal or no expression on normal tissues. This selective expression minimizes the risk of off-tumor effects and ensures that the therapy directly attacks cancer cells. Second, the target should play an essential role in PCa cell survival or proliferation. Its inhibition would interfere with key oncogenic pathways, thereby suppressing tumor growth. Third, the ideal antigen should be enriched within the PCa stem cell population, which drives tumor initiation, progression, and recurrence. Targeting these cells could yield more durable therapeutic outcomes.

Given these stringent criteria, a comprehensive understanding of the surface molecules expressed on PCa cells is crucial. Such knowledge provides the foundation for designing highly selective and potent CARs tailored to combat PCa effectively.

Among the various antigens explored in PCa (Table 1), PSMA and PSCA remain the most clinically advanced and well-validated, which justifies the greater focus on these targets in this review. Nonetheless, other antigens such as STEAP1/2, KLK2, EpCAM, B7-H3, nfP2X7, NKG2DL, F77 are emerging as promising candidates for CAR-T cell therapy, as they are predominantly expressed in PCa and associated with tumor proliferation and immune evasion.

Moreover, emerging evidence suggests that additional antigens may soon be validated as promising targets, potentially expanding the arsenal of CAR T cell therapies available for treating this disease. For example, recent evidence indicates that sirtuins, a family of NAD⁺-dependent deacetylases, play key roles in prostate cancer progression, metabolic regulation, and immune evasion [170]. Their modulation of pathways such as p53, NF-κB, and androgen receptor signaling may influence tumor immunogenicity and responsiveness to CAR-T therapies, warranting further exploration as potential co-targets in combinatorial immunotherapy.

Below, current research on surface antigens that have minimal expression outside the prostate and PCa lesions is summarized. These targets have either been used in preclinical studies or are already in clinical development for CAR T cell-based therapies against PCa (Table 2 and Table 3). Regarding clinical trials, it is important to note that most available CAR-T cell studies in PCa remain early-phase and involve limited cohorts, often enrolling with fewer than 20 patients per arm. Such small sample sizes preclude robust statistical analyses and restrict the generalizability of the results. Moreover, heterogeneous eligibility criteria and variations in lymphodepletion regimens further complicate cross-trial comparisons. Future multicenter studies employing standardized protocols and larger patient populations will be crucial to validate safety, efficacy, and optimal dosing parameters.

#### 3.2.1. Prostate-Specific Membrane Antigen (PSMA)

PSMA, also known as folate hydrolase 1, is a type II transmembrane glycoprotein composed of an extracellular ligand-binding domain, a transmembrane region, and a short intracellular tail.

PSMA functions as a glutamate-preferring carboxypeptidase. It is expressed at low levels in salivary glands, proximal kidney tubules, and duodenum, where it regulates folate metabolism [171]. It is also present in the central nervous system where modulates glutamate signaling [172].

Although normal prostate tissue expresses PSMA at low levels, its expression in PCa can increase by 100- to 1000-fold [173]. This upregulation correlates directly with high Gleason scores, advanced tumor stages, and progression to castration-resistant disease [174]. The folate hydrolase activity of PSMA enhances free folate uptake, thereby promoting tumor cell growth [175]. Curiously, PSMA expression is also observed in the tumor neovasculature of several non-prostatic cancers [176].

From a therapeutic perspective, the selective overexpression of PSMA in malignant prostate tissue, along with its apical localization in normal epithelial cells [177], offers potential to reduce on-target off-tumor toxicity. However, clinical trials of PSMA-targeted radionuclide therapy have reported cases of salivary gland hypofunction and xerostomia [178].

The strong diagnostic and therapeutic potential of PSMA is underscored by the success of PSMA-targeting radiopharmaceuticals recently approved by the FDA for both diagnosis and treatment of PCa. These agents feature a glutamate–urea–lysine core acting as a PSMA substrate surrogate, a pharmacokinetic-modulating linker, and a chelator group for radionuclide coordination [179]. Radiotracers approved for positron emission tomography (PET) imaging of PCa include [^68^Ga] Ga-PSMA-11 [180], [^18^F]DCFPyL [181], and [^18^F]F-rhPSMA-7.3 [182]. The β-emitting radiopharmaceutical [^177^Lu]Lu-PSMA-617 has shown marked antitumor activity in mCRPC. In the phase III VISION trial, patients treated with this agent experienced notably longer progression-free and overall survival, as well as delayed skeletal events and improved quality of life. These results led to its FDA approval for treating adults with PSMA-positive mCRPC [183]. Ongoing studies are further exploring novel radioligand-based therapies in various PCa settings [184]. Recently, the development of radiolabeled heterobivalent ligands targeting PSMA and other PCa-overexpressed antigens have shown promise in overcoming tumor heterogeneity [185,186,187,188].

PSMA-targeted strategies also include antibody–drug conjugates (ADCs) carrying cytotoxic payloads, such as the microtubule-targeting agents maytansinoid-1 (DM1) and monomethyl auristatin E (MMAE), which are currently under investigation [189]. The early success of PSMA-targeted radioligand therapies and ADCs highlights the potential for integrating PSMA into innovative T-cell redirecting therapies.

In the first preclinical study on 2nd-generation anti-PSMA CAR-T cell therapy, Maher et al. demonstrated that combining CD3ζ and CD28 signaling domains into the CAR construct effectively redirected and amplified human T-cell responses [190]. These pioneering results established the foundation for developing genetically engineered T cells with sustained cytotoxic activity against prostate tumor antigens. Building on these findings, a phase I dose-escalation trial (NCT01140373) was initiated to assess safety, optimal dosage, and targeting efficiency of the same anti-PSMA CAR-T cell construct. In this trial, patients received three escalating dose levels. Those in the higher dose cohorts experienced fevers and elevated interleukin levels, indicative of robust T-cell activation. CAR-T cells remained detectable in the blood for approximately two weeks [191].

In other studies, 1st-generation PSMA-targeted CAR-T cells successfully eliminated prostate cancer in various tumor models [192,193]. Sustained T-cell persistence for at least one week was required to achieve durable remissions, even without additional in vivo costimulation. These findings supported the progression to phase I clinical trial in patients with metastatic PCa. In this trial (NCT00664196), patients received chemotherapy conditioning followed by PSMA-targeted CAR-T cell infusion with continuous low-dose IL-2 [194]. Conditioning enhanced T-cell expansion and engraftment. Unexpectedly, higher engraftment levels were associated with an up to 20-fold depletion of IL-2. No anti-PSMA toxicities or anti-CAR immune responses were detected; nonetheless the study was suspended due to a lack of funding.

Preclinical studies have explored how structural modifications of PSMA-directed CAR-T cells influence their efficacy, both as monotherapies and in combination with other treatments. For example, Ma et al. used a γ-irradiation animal model to demonstrate the importance of nonmyeloablative preconditioning, showing that 2nd-generation CAR-T cells significantly outperformed 1st-generation design [195].

In another noteworthy investigation, Alzubi et al. engineered an innovative anti-PSMA CAR derived from a D7 single-chain antibody fragment and paired it with non-ablative, low-dose docetaxel [196]. This combination markedly suppressed tumor growth in murine models compared with CAR-T therapy alone. The authors also compared CAR-T cells engineered with CD28 or 4-1BB costimulatory domains. CD28-based CAR-T cells induced faster and more pronounced tumor regression, while 4-1BB-based CAR-T cells showed weaker activation, resulting in reduced differentiation and exhaustion [196]. Other preclinical studies have demonstrated that 3rd-generation CAR-T cells incorporating both CD28 and 4-1BB costimulatory domains can synergistically enhance antitumor activity compared with constructs containing a single costimulatory signal [130]. However, research by Zuccolotto et al. challenges this concept, demonstrating that constructs containing only the CD28 domain outperformed those engineered with both CD28 and 4-1BB [197,198]. The authors suggest that the addition of 4-1BB may be detrimental by promoting increased activation-induced cell death (AICD).

Collectively, these findings highlight the complexity of costimulatory domain selection in CAR design. While 4-1BB can improve persistence and reduce exhaustion in certain contexts, its combination with CD28 does not always yield synergistic benefits. The optimal configuration may therefore depend on the specific TME, antigen density, and therapeutic context, emphasizing the need for tailored CAR architectures to balance activation strength, persistence, and safety.

PCa is known to secrete TGF-β, which creates an immunosuppressive environment, promotes metastasis and neoangiogenesis, and strongly inhibits immune responses [199,200]. Increasing evidence shows that TGF-β not only suppresses CD8⁺ effector T-cell activity but also drives the conversion of CD4⁺ helper T cells into regulatory T cells (Tregs), collectively dampening anti-tumor immune responses [201]. To overcome these effects and improve CAR-T cell therapy, researchers have explored co-expressing a dominant-negative TGF-β receptor II (dnTGF-β RII), which lacks the intracellular kinase signaling domain [202,203], with an anti-PSMA CAR. This strategy aims to enhance T-cell infiltration, proliferation, persistence, and overall therapeutic efficacy against PCa [137,204].

Zhang et al. developed a 1st-generation anti-PSMA CAR construct incorporating both the CD3ζ gene and the dnTGF-β RII gene [204]. This construct effectively induces CD8+ T cells to become PSMA-reactive while rendering them resistant to the suppressive effects of TGF-β. In vitro studies demonstrated that PSMA-specific, TGF-β–resistant CD8⁺ T cells from mCRPC patients can be robustly expanded and effectively counteract the immunosuppressive TME, exhibiting significant cytotoxicity against PSMA-expressing tumor cell lines.

Kloss et al. designed an armored anti-PSMA CAR construct combining the 4-1BB costimulatory domain with the CD3ζ signaling domain, linked via a T2A element to dnTGF-β RII [137]. This construct enhanced T-cell proliferation, cytokine secretion, resistance to exhaustion and long-term in vivo persistence. In aggressive human prostate cancer mouse models, these armored CAR-T cells achieved robust and durable tumor regression. Based on these promising preclinical results, a phase I clinical trial (NCT03089203) is underway to assess the safety and feasibility of these CAR-T cells in patients with mCRPCa [205]. 30% of patients developed at least grade 2 CRS. In one case, massive clonal CAR T cell expansion caused a >98% drop in PSA but was associated with fatal sepsis. Acute surges in inflammatory cytokines were linked to these high-grade, yet manageable, CRS episodes. While some patients achieved a ≥30% reduction in PSA, CAR T-cell failure in others was linked to the upregulation of several inhibitory molecules in the TME. CAR T cell tracking demonstrated robust expansion in the bloodstream and effective tumor infiltration [206].

Despite the remarkable preclinical efficacy observed in murine models, the translation of armored anti-PSMA CAR-T cells to clinical settings has proven challenging. This discrepancy reflects fundamental differences between animal models and human tumors, emphasizing the need for better models and combination strategies to enhance outcomes.

Another multicenter phase I trial was initiated by the same group (NCT04227275) to further evaluate preliminary safety and efficacy. However, the study was terminated following severe safety events, (i.e., grade 5 events of immune-effector cell-associated neurotoxicity syndrome and multiorgan failure) and evidence of biological activity without sustained clinical response [207,208,209].

In later studies on anti-PSMA dnTGFβRII CAR-T cells, Weimin et al. engineered PSMA-targeted CAR-T cells to constitutively express an innovative inverted chimeric cytokine receptor (ICR), created by fusing the extracellular domain of the TGF-β receptor with the intracellular domain of the IL-7 receptor [210]. IL-7 is known to promote the survival of tumor-specific T cells [211], and preclinical studies have shown that genetically modification to enhance IL-7 signaling, either through secretion or receptor overexpression, can significantly boost their anti-tumor efficacy [212].

These modified PSMA-CAR-T cells constitutively expressing ICR exhibited markedly improved cytotoxicity and prolonged survival in mice compared with conventional PSMA-CAR-T cells, suggesting a promising new strategy for PCa treatment.

Other clinical studies (NCT05489991, NCT06046040) have investigated anti-PSMA CAR T-cells (TmPSMA-02) engineered with a CD2 endodomain and dual armoring provided by dnTGF-β RII and a PD-1:CD28 switch receptor. This fusion protein combines the extracellular ligand-binding domain of PD-1 with the transmembrane and cytoplasmic costimulatory domains of CD28. Upon PD-L1 binding, it boosts T cell activation, promoting cytokine release, proliferation, and cytotoxicity. Preliminary results confirmed the feasibility of producing the TmPSMA-02 construct. It exhibited potent cytotoxic activity, a favorable memory phenotype, and robust activation, while minimizing off-target immune responses. However, substantial toxicity related to macrophage activation syndrome (MAS) was observed [213,214]. As a result, study NCT05489991 was discontinued at the sponsor’s discretion [213].

Recent findings highlight the crucial role of TME in CRPC development. Myeloid-derived suppressor cells (MDSCs) secrete IL-23, which activates the androgen receptor pathway in PCa cells, promoting survival and proliferation even in low-androgen conditions [215]. Building on this insight, Wang et al. designed several CAR constructs including: (*i*) a duo-CAR by coexpressing IL-23 and PSMA specific CARs, (*ii*) a bispecific CAR by linking IL-23 and PSMA mAbs in a single construct, (*iii*) PSMA-CAR with soluble IL-23 mAb, and compared them with a standard PSMA-CAR [216]. The duo-CAR (IL-23mAb–4-1BB–CD3ζ–T2A–PSMAmAb–4-1BB–CD3ζ) induced stronger T cell activation and higher cytokine production than the bispecific CAR, despite comparable binding abilities. These results highlight that the CAR architecture, rather than binding affinity, plays a key role in T-cell persistence and sustained biological activity in vivo.

Another strategy for developing novel CAR constructs involves the use of camelid single-domain antibodies (nanobodies, V_H_H) [217]. Their high homology with the human VH family allows the design of CARs with reduced immunogenicity, minimizing the risk of adverse immune responses [218]. The small size of nanobodies also facilitates efficient cloning, transfection, and expression while preserving high antibody-like affinity. Nanobody-based CAR-T cells targeting PSMA have been developed and tested in PCa cell lines [219]. These constructs showed robust antigen-dependent expansion, increased IL-2 secretion, and overexpression of the activation marker CD69. Although these promising in vitro results, further preclinical studies are required to fully assess the therapeutic potential of V_H_H-based CAR-T cells in PCa.

LIGHT-PSMA CAR-T cells represent an innovative CAR-T cell therapy designed to enhance PCa treatment by targeting PSMA while co-expressing the LIGHT protein [220]. LIGHT, also known as tumor necrosis factor superfamily member 14 (TNFSF14), is expressed on activated T cells, natural killer cells, and immature dendritic cells [221,222]. It contributes to the reconstitution of tertiary lymphoid structures within the TME by promoting vasculature normalization and high endothelial venule formation.

Incorporation of LIGHT aims to remodel the TME, improving T-cell trafficking and cytotoxicity. In preclinical studies, a conventional 2nd-generation CAR-T cell construct, scFv(J591)–4-1BB–CD3ζ, was compared with an enhanced version co-expressing a modified LIGHT protein. This truncated form lacks cytoplasmic and transmembrane domains and is fused to a vascular targeting peptide (VTP), generating the scFv(J591)–4-1BB–CD3ζ–T2A–LIGHT-VTP construct. The modified construct demonstrated superior antitumor efficacy by combining targeted cytotoxicity with vascular and immunomodulatory effects [220]. In murine PCa models, LIGHT-secreting CAR-T cells significantly prolonged survival, increased immune cell infiltration, and enhanced chemokine expression without notable tissue damage, indicating good safety profile. A Phase I clinical trial (NCT04053062) started to evaluate the safety and efficacy of intravenously administered LIGHT-PSMA-CAR-T cells in patients with CRPC. However, this study was later suspended following efficacy assessment [223].

P-PSMA-101 is a CAR-T cell therapy specifically targeting PSMA, developed by Poseida Therapeutics for the treatment of mCRPC [224]. This autologous therapy is enriched for stem cell memory T cells (T_SCM_) and generated using the *piggyBac* non-viral transposon system. The construct encodes a proprietary Centyrin-based CAR (CARTyrin) that binds specifically to PSMA and includes a human-derived iCas9 safety switch activated by rimiducid to eliminate CAR-T cells if required. CARTyrin, a non-immunoglobulin scaffold, is designed to reduce immunotoxicity compared with antibody-derived scFv CARs, improving persistence and limiting T-cell exhaustion. The high percentage of T_SCM_ cells contributes to enhanced efficacy, safety, and bone tropism, critical factors in PCa therapy. P-PSMA-101 showed marked tumor regression in preclinical murine models of PCa providing a rationale for clinical evaluation.

A Phase I clinical trial (NCT04249947) was subsequently launched to assess safety and efficacy of P-PSMA-101 in patients with mCRPC [224,225]. Early findings showed encouraging but preliminary clinical responses at low doses in heavily pretreated patients, along with a favorable safety profile. CRS was manageable, and no neurotoxicity was reported. As for the latest updates, the Phase I trial of P-PSMA-101 has been terminated. These results highlight the ongoing challenge of translating strong preclinical efficacy into meaningful clinical benefit in solid tumors due to antigen heterogeneity, immune evasion, and safety limitations.

Preclinical studies showed that combining PSMA-directed CAR T-cell therapy with PD-1 blockade enhances antitumor activity. For instance, anti-PD-1 antibodies used alongside 2nd-generation PSMA-targeted CAR T cells improved treatment outcomes in PCa models [226].

A Phase I clinical trial (NCT04768608) evaluated the safety and efficacy of non-virally engineered, PD-1-knockout anti-PSMA CAR T cells in patients with refractory CRPC. In this trial, CAR T cells were generated via CRISPR/Cas9 to delete PD-1 and integrate anti-PSMA constructs, with the goal of overcoming the immunosuppressive TME of CRPC. The trial has been completed, but detailed safety and efficacy results have not yet been released [227].

PD-1 silent PSMA/PSCA CAR-T cells represent another innovative strategy for PCa therapy. These engineered lymphocytes simultaneously target two key antigens: PSMA and prostate stem cell antigen (PSCA). This dual-targeting approach enhances recognition and elimination of tumor cells expressing either or both antigens, thereby addressing tumor heterogeneity and reducing the risk of antigen escape. To further boost therapeutic efficacy, the CAR-T cells are designed to silence the PD-1 receptor, thereby attenuating the immunosuppressive effects of the TME. A Phase I clinical trial (NCT05732948) is currently evaluating the safety and efficacy of these PD-1-silent PSMA/PSCA-targeted CAR-T cells in patients with mCRPC [228].

Shenzhen Geno-Immune Medical Institute has developed an innovative CAR platform, termed 4SCAR, which incorporates multiple costimulatory domains along with an inducible suicide gene (CD28–CD27–CD3ζ–T2A–iCas9) [229]. This advanced design provides strong antitumor activity while significantly mitigating the risk of CRS. Two phase I clinical trials are currently assessing the safety and efficacy of 4SCAR-T cells. One study (NCT04429451) focuses exclusively on PSMA-targeted therapy [230], while a second trial (NCT05437315) explores a bispecific approach targeting both disialoganglioside (GD2) and PSMA [231]. GD2 is a well-established tumor-associated antigen that drives oncogenic signaling pathways, enhancing cell proliferation, migration, and invasion. Notably, GD2 is expressed in a subset of PCa cells, with higher levels observed in metastatic tumors [232]. Both trials are currently recruiting participants.

AvenCell has recently developed UniCAR02-T, a universal, switchable CAR-T platform designed to enhance safety and control. In this system, T cells remain inactive until administration of the targeting module TMpPSMA, a recombinant PSMA-specific antibody fused to a peptide epitope recognized by UniCAR02-T [233]. TMpPSMA simultaneously binds PSMA on prostate cancer cells and engages UniCAR on T cells, thereby triggering targeted cytotoxicity. Discontinuing TMpPSMA quickly deactivates UniCAR-T cells, reducing risks of CRS and neurotoxicity. Safety, tolerability, and therapeutic potential of UniCAR02-T cells combined with TMpPSMA have been investigated in a Phase I clinical trial (NCT04633148) in patients with progressive CRPC. Although the therapy was well tolerated, it demonstrated limited biological efficacy, leading to the discontinuation of further development [234]. The trial was terminated during the dose-escalation phase due to limited patient recruitment and lack of efficacy signals. The UniCAR02-T platform introduces a controllable “on/off” switch to improve CAR-T safety and flexibility. However, its limited efficacy in CRPC underscores the ongoing challenges of translating CAR-T therapy to solid tumors. Future refinements may focus on improving tumor infiltration and optimizing the pharmacokinetics of the targeting modules to fully realize the potential of modular CAR-T systems.

NT-1921 is an innovative immunotherapy developed by Nova Therapeutics LLC to specifically target mCRPC [235]. Powered by Nova’s proprietary *nSMART* technology, NT-1921 utilizes an advanced CAR-T cell platform to modulate the TME. By enhancing TILs activity and reducing immunosuppressive Tregs within the TME, NT-1921 aims to promote CAR-T cells proliferation and persistence while minimizing systemic toxicity. Preliminary Phase I trial data (NCT05656573) [236] indicate a favorable safety profile alongside promising efficacy outcomes. Beyond direct cytotoxicity through cytokine release and accumulation in the TME, NT-1921 may also resensitize mCRPC patients to first-line antiandrogens like enzalutamide or apalutamide.

Two additional early-phase clinical trials, NCT05354375 [237] and NCT06228404 [238] are evaluating enhanced PSMA-targeted CAR-T cell therapies in CRPC. Both trials are currently recruiting participants. Specific details about the CAR constructs remain undisclosed in public sources.

#### 3.2.2. Prostate Stem Cell Antigen (PSCA)

Prostate stem cell antigen (PSCA) is a member of Thy-1/Ly-6 family of glycosylphosphatidylinositol (GPI)-anchored proteins. Despite its name, PSCA is not a specific marker for stem cells, nor is it exclusively expressed in the prostate, as it is also abundantly found on epithelial cells of the bladder, kidney, esophagus, and stomach [239]. Although the precise physiological and pathological roles of PSCA remain unclear [240], other GPI-anchored proteins within the same family are implicated in T cell activation, proliferation, stem cell survival, and responses to cytokines and growth factors [241,242]. They have also been implicated in carcinogenesis [243] and in promoting tumor cell adhesion [244].

PSCA is overexpressed in more than 90% prostate cancer [245] and its expression correlates with Gleason score, clinical stage, invasion, metastasis and androgen independence [245,246,247]. PSCA is also expressed in other tumors, including gastric, bladder and pancreatic cancers [248]. Collectively, these features make PSCA a promising antigen target for CAR-T cell therapy.

Preclinical studies employing both xenograft and syngeneic tumor models, have shown that 2nd-generation PSCA-specific CAR-T cells, incorporating a 4-1BB costimulatory domain alongside CD3ζ signaling, exhibit robust and sustained antitumor responses in multiple cancer settings, including bone metastatic prostate cancer [249]. Notably, PSCA-CARs containing 4-1BB motif exhibit superior antigen specificity and reduced T-cell exhaustion while maintaining tumor-killing potency comparable to CD28-based CARs. Cyclophosphamide preconditioning significantly reshapes the TME by reducing immunosuppression, promoting proinflammatory signals, and increasing recruitment of antigen-presenting cells. These combined effects sustain antitumor immunity and markedly improve the therapeutic potential of PSCA-targeted CAR T cells [250].

Building on these promising results, the same CAR-T cells were evaluated in a Phase I clinical trial (NCT03873805) in patients with mCRPC to assess safety and bioactivity. The study demonstrated biological activity and early clinical benefit, although cystitis, likely an on-target/off-tumor effect exacerbated by cyclophosphamide lymphodepletion, was observed [251]. These encouraging antitumor responses and the overall favorable toxicity profile have driven the ongoing Phase Ib trial (NCT05805371), which aims to improve T cell persistence and boost therapeutic efficacy [252]. These findings highlight the potential of PSCA-targeted CAR-T therapy in PCa while underscoring the need to carefully balance efficacy with on-target/off-tumor toxicity. Optimizing CAR design, lymphodepletion strategies, and patient selection will be critical to translating preclinical promise into sustained clinical benefit.

Bellicum Pharmaceuticals engineered a first-generation anti-PSCA CAR coexpressing an inducible dual costimulatory module (iMC), which links two FKBP12-F36V binding proteins in-frame with the MyD88 and CD40 signaling domains [253]. MyD88 is the principal adaptor for Toll-like receptors and IL-1 receptor signaling, while CD40 is a T cell costimulatory receptor essential for memory formation. Inducible activation of MyD88 and CD40 with the dimerization-inducer rimiducid, stimulates these pathways, boosting CAR-T expansion, cytokine secretion, and persistence. This on/off system aims to improve antitumor activity while minimizing constitutive toxicity by restricting signaling to periods of intentional drug dosing [254,255].

A Phase I/II clinical trial (NCT02744287) evaluated the safety and activity of this investigational CAR-T therapy, BPX-601, in patients with PSCA-positive mCRPC [256]. Despite encouraging interim data, Bellicum Pharmaceuticals announced on 15 March 2023 that the trial would be discontinued due to severe immune-mediated adverse events, including grade 4 CRS [257], underscoring the persistent challenge of balancing efficacy and safety in CAR-T therapy for solid tumors.

#### 3.2.3. Six-Transmembrane Epithelial Antigen of Prostate 1 (STEAP1)

The six transmembrane epithelial antigen of the prostate (STEAP) family includes four proteins, STEAP1 to STEAP4, originally identified in the prostate tissue [258]. All members share a common structure with six transmembrane domains and both intra- and extracellular loops, suggesting potential roles as channels or transporters [259]. Their similarity to metalloreductases indicates possible involvement in iron and copper reduction [260]. Beyond metal metabolism, STEAP proteins are associated with key cellular processes such as proliferation, invasion, apoptosis, oxidative stress, and inflammation [261,262,263,264].

Discovered in 1999 as a prostate-specific cell-surface antigen, STEAP1 is markedly overexpressed in PCa [265]. It plays a crucial role in metal ion metabolism and cellular communication. Moreover, STEAP1 promotes cancer cell proliferation, invasion, and epithelial-to-mesenchymal transition [265,266,267].

High STEAP1 expression in over 80% of mCRPC cases, combined with its limited presence in normal human tissues [265,268], makes it an attractive therapeutic target in PCa [269]. On these bases, 2nd-generation STEAP1-targeted (STEAP1-BBζ) CAR-T cells have been engineered and evaluated for antitumor activity in PCa models [270]. Preclinical experiments using a human STEAP1 knock-in mouse model demonstrated that STEAP1-BBζ CAR-T cells maintain significant antitumor activity even under low antigen density [270]. Resistance due to STEAP1 antigen escape can be overcome by combination treatment with IL-12 delivered as a collagen-binding domain fusion protein (CBD–IL-12) [270]. These promising findings have paved the way for a first-in-human phase I/II clinical trial (NCT06236139). The trial is evaluating STEAP1 CAR T cell therapy in combination with enzalutamide in patients with mCRPC, to assess both safety profile and antitumor efficacy [271].

#### 3.2.4. Six-Transmembrane Epithelial Antigen of Prostate 2 (STEAP2)

STEAP2, also known as six transmembrane protein of prostate 1 (STAMP1), is another member of the STEAP family primarily expressed in prostatic tissue [272,273]. Notable expression also occurs in the brain, pancreas, ovary, and broadly in neuronal tissue [258]. Its higher expression in PCa compared with normal tissues make it a promising therapeutic target [274,275]. Researchers at Astra Zeneca developed AZD0754, a CAR-T cell therapy targeting STEAP2. These CAR-T cells are armored with a dominant-negative TGF-β type II receptor to enhance efficacy in the immunosuppressive PCa microenvironment [276]. AZD0754 exhibited potent and specific cytotoxicity against STEAP2-expressing cells in vitro, even under TGF-β-rich conditions. In xenograft mouse models, it demonstrated robust, dose-dependent antitumor activity against tumors derived from both STEAP2-expressing cancer cell lines and patient-derived tumors. Preclinical safety data support AZD0754 as a potential first-in-class CAR T therapy for PCa. A first-in-human, multicenter, open-label Phase I/II clinical trial (NCT06267729) is now underway. This trial will evaluate safety, cellular kinetics, pharmacodynamics, preliminary efficacy, and manufacturing feasibility of AZD0754 in patients with mPCa [277].

#### 3.2.5. Epithelial Cell Adhesion Molecule (EpCAM)

Epithelial cell adhesion molecule (EpCAM), also known as CD326, is a type I transmembrane glycoprotein expressed on the surface of epithelial cells, where it regulates cell adhesion, proliferation, migration, and differentiation [278]. EpCAM is overexpressed in prostate cancer tissues and metastases [279,280]. Moreover, it is considered a cancer stem cell marker, further supporting its potential as a therapeutic target [281].

A 2nd-generation EpCAM-specific CAR was constructed using an anti-EpCAM scFv fused to the CD28 and CD3ζ endodomain. Preclinical studies in mouse xenograft models have demonstrated the antitumor efficacy of these EpCAM-targeting CAR T-cells [282]. Their administration significantly inhibits tumor growth, likely through targeting the cancer stem cell compartment. However, the expression of EpCAM on normal epithelial tissues, such as lung tissue, raises safety concerns. In one study, mice treated with 3rd-generation EpCAM-CAR T-cells [scFv(G8.8)–CD28–4-1BB–CD3ζ] developed severe pulmonary immunopathology, underscoring the need for comprehensive preclinical toxicity evaluations before clinical application [283].

Nonetheless, phase I/II clinical trials are currently evaluating the safety and efficacy of 2nd-generation EpCAM CAR-T cell therapy in several cancers, including PCa (NCT03013712). Patients are being monitored for up to 24 months to assess adverse events, persistence of CAR-T cells and therapeutic efficacy [284].

#### 3.2.6. Kallikrein-Related Peptidase 2 (KLK2)

Kallikrein-related peptidase 2 (KLK2) is a serine protease belonging to the kallikrein family of enzymes. It is predominantly expressed in prostate tissues, often coexpressed with other kallikreins such as KLK3, which is also known as PSA [285]. Elevated levels of KLK2 have been associated with the PCa development and progression [286,287]. The enzyme’s activity may contribute to tumor growth and metastasis, positioning KLK2 as both a diagnostic marker and a potential target for immunotherapeutic approaches, including CAR-T cells [288].

JNJ-75229414 is an investigational CAR-T cell therapy specifically targeting KLK2 and is currently being evaluated in early-phase clinical trials (NCT05022849) to evaluate its safety, efficacy, and tolerability in patients with mCRPC [289]. More recently, Janssen and Fate Therapeutics have developed an off-the-shelf CAR-T cell product targeting KLK2, using induced pluripotent stem cell (iPSC) technology [290,291]. A clonal master iPSC line containing the TRAC-edits was then differentiated into T cells (CAR-KLK2 iT cells) that do not express the T-cell receptor, thereby eliminating the risk of graft-versus-host disease. Preclinical studies demonstrated that CAR-KLK2 iT cells could selectively target prostate cancer cells, although further optimization is required before clinical translation.

#### 3.2.7. Type I Transmembrane Protein B7-H3 (CD276)

As outlined in Section 2.1, the development of agents that enhance T cell–mediated antitumor immunity through immune checkpoint blockade has markedly expanded the clinical landscape of cancer immunotherapy [40]. The type I transmembrane protein B7-H3 (CD276), an immune checkpoint molecule belonging to the B7 family, is abundantly expressed on cancer cells and activated, tumor-infiltrating immune cells. By engaging inhibitory signaling pathways, B7-H3 enables tumors to escape destruction by cytotoxic T lymphocytes and natural killer cells [292]. Consequently, it has emerged as a highly attractive target for novel anticancer therapy [293]. B7-H3 is upregulated in PCa, though it is not a strictly tumor-specific antigen [294]. In prostate tumors, high B7-H3 expression correlates with immune evasion, enhanced tumor aggressiveness, increased metastatic potential, higher recurrence rates, and poorer clinical outcomes [292]. Li et al. engineered a 2nd-generation CAR specifically targeting B7-H3 that incorporates CD28 as a costimulatory domain. This construct potently inhibited prostate tumor growth in vitro and in vivo in an antigen-dependent manner [295]. Furthermore, exposure to B7-H3–positive tumor cells triggered robust CAR-T cell expansion and secretion of key cytokines, including IFN-γ and TNF-α. Collectively, these findings validate B7-H3 as a promising therapeutic target in PCa and strongly support further clinical development of B7-H3-specific CAR-T cell therapies.

#### 3.2.8. Non-Functional P2X Purinoceptor 7 (nfP2X7)

P2X purinoceptor 7 (P2X7) is an ATP-gated ion channel expressed on the cell surface. A dysfunctional variant of this receptor, termed non-functional P2X7 (nfP2X7), has emerged as a promising target for CAR-T cell therapy because it is exclusively expressed on cancer cells and antigenically distinct from the functional receptor present on healthy cells [296,297,298].

The nfP2X7-CAR-T cells specifically target the P2X7 receptor gene locus, enabling a pancancer therapeutic strategy with high on-target specificity and minimal off-target cytotoxicity [299]. Notably, nfP2X7-directed CAR-T cells incorporating the 4-1BB costimulatory domain and CD3ζ signaling module have demonstrated potent antitumor efficacy in xenograft mouse models of PCa and exhibited cytotoxicity against several solid cancer cell lines. These findings highlight that nfP2X7-targeted CAR-T cells as a potential broad-spectrum immunotherapeutic approach for treating solid malignancies in humans [299].

#### 3.2.9. Natural Killer Group 2 Member D Ligand (NKG2DL)

Ligands for the Natural Killer Group 2 Member D (NKG2D) receptor, an activating receptor expressed on NK cells [300], have emerged as broadly applicable tumor-associated markers and, consequently, attractive targets for CAR-T cell therapy. These stress-induced ligands are upregulated in a wide range of malignancies, including PCa, while remaining virtually undetectable or only minimally expressed on healthy tissues [301]. This tumor-restricted expression profile makes NKG2D ligands ideal candidates for selective immunotherapeutic intervention.

He et al. developed two distinct 2nd-generation NKG2D-based CAR-T cell constructs. The first, NKG2D-CAR, features the extracellular domain of human NKG2D fused to 4-1BB and CD3ζ signaling domains. The second construct, NKG2DIL7-CAR, additionally incorporates the human IL-7 gene, linked to the CD3ζ signaling domain via a T2A ribosomal skipping sequence. Data from xenograft mouse models of human prostate tumors demonstrates that NKG2D-targeted CAR T cells significantly inhibited tumor growth and prolonged survival of treated animals. In vitro and in vivo studies showed that these CAR T cells exert markedly increased cytotoxicity against PCa compared to non-transduced T cells [302]. Furthermore, coexpression of IL-7 further improved antitumor efficacy by reducing T cell exhaustion, maintaining a less-differentiated cell phenotype, boosting antigen-dependent cytokine production, and reducing apoptosis. Together, these effects improve the persistence and survival of the CAR T cells [302].

#### 3.2.10. F77 Antigen

F77, originally identified as the prostate cancer lipid antigen (PCLA), is a unique carbohydrate antigen expressed on both androgen-dependent and -independent PCa cells, making it an attractive target for immunotherapy [303]. Leveraging a murine F77-specific mAb, researchers have engineered 2nd-generation CAR-T cells bearing an scFv directed against F77 and either CD28 or 4-1BB costimulatory domains [304]. In preclinical models, these F77-targeted CAR-T cells demonstrated robust, antigen-dependent cytotoxicity and achieved complete eradication of PC3 prostate tumors in human xenograft models. Collectively, these findings underscore F77’s potential as a versatile immunotherapeutic target in PCa, and potentially in other F77-expressing malignancies.

## 4. Conclusions and Future Perspectives

CAR-T cell therapy has revolutionized the treatment of hematologic malignancies, whereas it remains challenging in solid tumors due to multiple physical, immunological, and molecular barriers that limit its efficacy [102,135]. In PCa, these barriers include a dense extracellular matrix and a disorganized vascular network, which impede CAR-T cell infiltration into the tumor site. The TME further suppresses T-cell activity through immunosuppressive cells, including MDSCs, Tregs, and tumor-associated macrophages, which secrete inhibitory cytokines such as TGF-β and IL-10. Local metabolic conditions, such as glucose depletion, acidic pH and hypoxia, create additional challenges that compromise CAR-T cell function and persistence [12,15,17]. Furthermore, the hostile TME can drive T-cell exhaustion and upregulation of immune checkpoints, limiting cytotoxic activity and the duration of antitumor activity, thereby making long-term disease control difficult [138].

Finally, although antigens like PSMA and PSCA are overexpressed on prostate tumor cells, their low-level expression in normal tissues poses a risk of on-target, off-tumor toxicity. Moreover, heterogeneity of antigen expression facilitates immune escape [305]. To address these challenges, several innovative strategies have been developed.

To counteract the immunosuppressive TME, armored CAR-T cells have been developed that incorporate cytokine “payloads” (e.g., IL-7, IL-12, IL-23) to remodel the TME by activating endogenous immune effectors and repolarizing MDSCs/M2-macrophages [210,216,270,302]. In other approaches, CAR-T cells have been engineered to resist TGF-β-mediated suppression (e.g., via a dominant-negative TGF-β receptor) or converting PD-1 engagement into an activating signal (via PD-1/CD28 switch receptor) [137,214]. These promising strategies allow to bypass checkpoint-mediated exhaustion. Preclinical studies have demonstrated improved functionality under immunosuppressive conditions; however, durable clinical responses still require confirmation in humans. However, they may also increase the risk of CRS if constitutive expression is not adequately regulated. Balancing potency with safety thus remains a critical challenge for next-generation CAR engineering. Also, combination with immune checkpoint inhibitors may counteract the hostile TME by reviving exhausted CAR-T cells in situ [139].

Recently, the CoupledCAR^®^ platform has been developed, harnessing two complementary CAR constructs, one targeting PAP and the other CD19, expressed on B-lymphocytes. This dual-CAR approach promotes robust T-cell expansion, enhances tumor infiltration, and boosts resistance to the immunosuppressive TME [306]. To address antigen heterogeneity and immune escape, dual antigen-targeting CARs (e.g., PSMA/PSCA or PSMA/GD2) have been engineered [228,231]. Bispecific CAR constructs can more effectively delay tumor escape compared to single-antigen CARs, although their increased complexity may raise manufacturing costs. While these designs improve tumor recognition and reduce relapse risk, their complex signaling integration can alter T-cell activation thresholds, potentially increasing exhaustion or off-tumor toxicity.

In this context, other strategies led to the development of universal or switchable CAR platforms (i.e., UniCAR) which employ adaptor molecules to flexibly retarget a single CAR backbone toward multiple antigens [160]. This modularity could, in theory, enable dynamic reassignment of CAR specificity if antigen downregulation occurs. However, clinical application remains challenging, as it requires continuous administration of adaptor molecules. Interestingly, coupling UniCAR T-cells with a target module conjugated to a radiolabeled ligand provides a novel immunotheranostic strategy for PCa [307,308]. Additional engineering refinements, such as inducible costimulation and safety switches, aim to enhance persistence without increasing toxicity. The iMC system pioneered by Bellicum Pharmaceuticals (e.g., BPX-601) demonstrates that small-molecule control over MyD88/CD40 signaling can amplify CAR-T expansion on demand and then be halted by withdrawing the dimerizer [253]. Likewise, suicide switches (e.g., iCas9) allow rapid elimination of CAR-T cells in cases of severe toxicity [150]. In both systems, defining optimal dosing schedules for dimerization inducers will be critical to achieve predictable pharmacokinetic profiles and safe clinical translation.

Finally, enriching for less-differentiated T-cell subsets, such as central memory or stem cell memory T-cells, prior to CAR transduction enhances proliferative capacity and long-term persistence. However, balancing robust in vivo expansion (necessary for antitumor effects) with safety (avoiding prolonged cytokine release) remains a delicate compromise.

In summary, CAR-T cell therapy for PCa is still in its early developmental stage, yet initial clinical trials have demonstrated its feasibility and justified cautious optimism. These pioneering investigations have shown that CAR-T cells can not only kill prostate tumor cells but also modulate the challenging TME. To translate this into a clinically effective therapy, next-generation strategies must overcome solid tumor-specific barriers through continued innovation in CAR design, optimized cell manufacturing, and rational combination approaches.

Next-generation CAR-T cell therapies are dedicating increasing amounts of focus to exploring allogeneic, ‘off-the-shelf’ products derived from healthy donors or iPSCs to overcome manufacturing delays and cost constraints associated with autologous approaches [309]. Genetic editing technologies (CRISPR/Cas9, TALENs) are used to eliminate endogenous TCR and MHC molecules, minimizing graft-versus-host disease while preserving antitumor potency [168]. Beyond genetic editing, metabolic engineering of CAR-T cells, such as promoting oxidative phosphorylation or modulating lipid metabolism, has emerged as a means to enhance persistence and resistance to the nutrient-deprived, hypoxic tumor microenvironment [310]. Similarly, co-expression of chemokine receptors (e.g., CXCR2, CCR5) improves trafficking toward chemokine-rich tumor sites [311].

Combining CAR-T cell therapy with complementary treatment modalities offers a promising approach to overcome the multifaceted barriers that limit efficacy in prostate cancer [312]. For instance, radioligand therapy directed against PSMA can reduce tumor burden and enhance antigen presentation, thereby sensitizing residual tumor cells to CAR-T-mediated cytotoxicity [313]. Likewise, the integration of CAR-T therapy with immune checkpoint inhibitors (e.g., anti-PD-1/PD-L1 or anti-CTLA-4 antibodies) has demonstrated synergistic effects by mitigating T-cell exhaustion and promoting sustained persistence [226]. In a similar vein, PARP inhibitors, by blocking DNA repair, can increase tumor DNA damage and mutational burden [312], potentially enhancing CAR-T cell cytotoxicity through an increased neoantigen load and the induction of immunogenic cell death. Collectively, these rational combination strategies hold the potential to enhance both the efficacy and durability of therapeutic responses in advanced PCa.

Future developments in this field will rely on a multidisciplinary synergy among immunology, bioengineering, oncology and computational biology, all working together to optimize the effectiveness of treatment. Although significant challenges remain, the rapidly advancing scientific research and expanding clinical experience suggest that CAR-T therapy may ultimately make a meaningful and transformative contribution to the management of prostate cancer.

## Figures and Tables

**Table 1:** Comparative overview of prostate cancer target antigens investigated for CAR-T cell therapy.

Target Antigen	Expression Profile	Advantages	Disadvantages/Limitations	Clinical Status
PSMA	Highly overexpressed in primary and metastatic PCa; low expression in normal prostate, kidneys, salivary glands, and small intestine.	Highly validated antigen; extensive preclinical/clinical data; strong tumor specificity; suitability for radiopharmaceutical development.	On-target/off-tumor toxicity (salivary glands, kidneys); antigen heterogeneity; immune escape.	Multiple phase I/II trials
PSCA	Overexpressed in 90% of PCa; also expressed in other tumors; low expression in normal prostate, bladder and pancreas.	Coexpression with PSMA allows dual targeting; promising preclinical safety.	Severe immune-mediated adverse events (i.e., CRS).	Phase I trials
STEAP1	Expressed in prostate, respiratory tract and CNS Markedly overexpressed in PCa and mCRPC.	Well-conserved across tumor stages; effective in combination with IL-12.	Limited clinical data	Phase I/II
STEAP2	Expressed in prostate, brain, pancreas, ovary; upregulated in PCa.	Increased potency in hostile TME through armored CAR-T	Limited clinical data; moderate expression in non-prostatic tissues.	Phase I/II
EpCAM	Broadly expressed in epithelial tumors including PCa; limited expression in normal epithelia.	Accessible surface antigen.	High risk of on-target/off-tumor effects	Early-phase studies
KLK2	Restricted to prostate epithelium and PCa; androgen-regulated enzyme.	Prostate-restricted; relevance to cancer progression.	Expression heterogeneity in advanced PCa.	Phase I
B7-H3	Overexpressed in PCa and many solid tumors; low in normal tissue.	Excellent immunotherapeutic target; favorable tumor-to-normal expression ratio.	Limited clinical validation in PCa; cross-reactivity with vasculature.	Preclinical.
nfP2X7	Aberrantly expressed on a broad range of malignancies	Unique cancer-specific epitope; not expressed in healthy tissues.	Early discovery stage; limited expression data in Protein Atlas.	Preclinical.
NKG2DL	Upregulated by stress, chronic inflammation, irradiation, or certain drug; expressed in PCa and TME	Broadly expressed on tumors and immunosuppressive cells	Expression on normal cells “on-target, off-tumor” toxicity risks.	Preclinical.
F77	Expressed on PCa cells; absent in normal tissue.	Highly tumor-specific; potential marker for aggressive PCa.	Limited validation; target epitope heterogeneity.	Preclinical.

**Table 2:** Clinical studies on anti-PSMA CAR–T cell therapy in PCa.

NCT Number	Target	CAR Construct	Intervention *	Tumor Type	Phase	N° of Patients	Outcome Measures	Status
NCT01140373	PSMA	scFv(J591)–CD28–CD3ζ	2nd-gen CAR-T cells	CMPC	I	13	DLT, CTC, PSA response, T-cell expansion/persistence	Active, not recruiting
NCT00664196	PSMA	scFv(3D8)–CD3ζ	1st-gen CAR-T cells IL–2	CRPC	I	18	AEs, MTD, Tumor response, PK, PD	Suspended
NCT03089203	PSMA	scFv(J591)–4-1BB–CD3ζ–T2A–dnTGFβRII	CART–PSMA–TGFβRDN cells +/− standard LD regimen	CRPC	I	23	TRAE, ORR (RECIST), PSA response, OS, PFS	Active, not recruiting
NCT04227275	PSMA	scFv(J591)–4-1BB–CD3ζ–T2A–dnTGFβRII	CART–PSMA–TGFβRDN cells anakinra	mCRPC	I	16	DLT, AEs, ORR (PSA response), T-cell expansion/persistence	Stopped
NCT05489991	PSMA	scFv(J591)–CD2–CD3ζ–T2A–dnTGFBR–T2A–PD1–CD28	TmPSMA–02 CAR-T cells	mCRPC	I/II	1	AEs, SAEs, RP2D, MTD, DLT, ORR (RECIST)	Stopped
NCT06046040	PSMA	scFv(J591)–CD2–CD3ζ–T2A–dnTGFBR–T2A–PD1–CD28	TmPSMA–02 CAR-T cells	mCRPC	I	18	AEs, DLT; MTD	Recruiting
NCT04053062	PSMA	scFv(J591)–4-1BB–CD3ζ–T2A–LIGHT-VTP	LIGHT–PSMA CAR-T cells	CRPC	I	12	AEs	Suspended
NCT04249947	PSMA	Undisclosed CAR	P–PSMA–101 CAR-T cells rimiducid	mCRPC	I	40	AEs, DLT, ORR (RECIST)	Terminated
NCT04768608	PSMA	Undisclosed CAR	PD1–PSMA–CART cells	CRPC	I	3	AEs, PSA response, ORR, T-cells expansion/persistence	Completed
NCT05732948	PSMA PSCA	Undisclosed CAR	PD–1 Silent PSMA/PSCA CAR-T cells	mCRPC	I	12	AEs, DLT	Recruiting
NCT04429451	PSMA	anti-PSMAscFv–CD28–CD27–CD3ζ–T2A–iCas9	4SCAR–PSMA T–cells rimiducid	PCa	I/II	100	AEs, DLT, ORR, PFS, OS, T-cells expansion/persistence	Recruiting
NCT05437315	PSMA GD2	anti–GD2/PSMAscFvs–CD28–CD27–CD3ζ–T2A–iCas9	bi–4SCAR–GD2/PSMA T–cells rimiducid	PCa	I/II	60	AEs, DLT, ORR, PFS, OS, T-cells expansion/persistence	Recruiting
NCT04633148	PSMA	Undisclosed CAR	UniCAR02–T TMpPSMA	CRPC	I	16	AEs, DLT, MTD, RP2D, ORR, DOR, PFS, OS, PSA response, CTC	Terminated
NCT05656573	PSMA	Undisclosed CAR	NT–1921	mCRPC	I	20	AEs, PFS, OS, CTC, cytokine profile, PSA response, T-cells expansion/persistence	Recruiting
NCT05354375	PSMA	Undisclosed CAR	PSMA–CAR-T cells	CRPC	I	20	AEs, ORR, PFS, OS	Recruiting
NCT06228404	PSMA	Undisclosed CAR	PSMA–CAR-T cells	refractory CRPC	I	18	DLT, PSA response, ORR	Recruiting

* Before each CAR-T infusion, patients undergo standard lymphodepletion regimen (cyclophosphamide + fludarabine). Abbreviations: AEs, Adverse Effects; CMPC, Castration-sensitive Metastatic Prostate Cancer; CRPC, Castration-Resistant Prostate Cancer; CTC, Circulating Tumor Cell; DLT, Dose Limiting Toxicity; DOR, Duration Of Response;; mCRPC, metastatic Castration-Resistant Prostate Cancer; MTD, Maximum Tolerated Dose; ORR, Objective Response Rate; OS, Overall Survival; PCa, Prostate Cancer; PD, Pharmacodynamics; PFS, Progression-Free Survival; PK, Pharmacokinetics; PSA, Prostate-Specific Antigen; RECIST, Response Evaluation Criteria In Solid Tumors; RP2D, Recommended Phase 2 Dose; SAEs, Serious Adverse Events; TRAE, treatment-related adverse event.

**Table 3:** Clinical studies on other antigens-targeted CAR–T cell therapy in PCa.

NCT Number	Target	CAR Construct	Intervention *	Tumor Type	Phase	N° of Patients	Outcome Measures	Status
NCT03873805	PSCA	scFv(hA11)–4-1BB–CD3ζ	PSCA CAR-T cells	mCRPC	I	14	AEs, DLT, RP2D, PFS, OS, cytokine profile, T-cells expansion/persistence	Active, not recruiting
NCT05805371	PSCA	scFv(hA11)–4-1BB–CD3ζ	PSCA CAR-T cells +/− radiation	mCRPC	Ib	21	AEs, DLT, RP2D, PFS, OS, PSA response, cytokine profile, T-cells expansion/persistence	Recruiting
NCT02744287	PSCA	iMC–T2A–scFv(hA11)–CD3ζ	BPX-601 rimiducid	mCRPC	I II	151	AEs, DLT, MTD, RP2D, PD, ORR (RECIST)	Suspended
NCT06236139	STEAP1	scFv(mAb120.545)–4-1BB–CD3ζ	STEAP1 CAR-T cells, enzalutamide	mCRPC	I II	48	AEs, ORR (RECIST) PSA response, TTR, DOR, PFS, OS	Recruiting
NCT06267729	STEAP2	scFv(40A3)–4-1BB–CD3ζ–T2A–dnTGFβRII	AZD0754	mPCa	I II	60	AEs, DLT, PSA response, ORR, TTR, DOR, PFS, OS	Recruiting
NCT03013712	EpCAM	Undisclosed CAR	EpCAM CAR-T cells	PCa	I II	60	AEs, ORR (RECIST), OS	Unknown status
NCT05022849	KLK2	Undisclosed CAR	JNJ–75229414 +/− Bridging therapy with anti-AR agents/docetaxel/radiotherapy	mCRPC	I	15	AEs, DLT, ORR, TTR, DOR, T–cell expansion/persistence	Active, not recruiting

* Before each CAR-T infusion, patients undergo standard lymphodepletion regimen (cyclophosphamide + fludarabine). Abbreviations: AEs, Adverse Effects; l; DLT, Dose Limiting Toxicity; DOR, Duration Of Response; mCRPC, metastatic Castration-Resistant Prostate Cancer; mPCa, metastatic Prostate Cancer; MTD, Maximum Tolerated Dose; ORR, Objective Response Rate; OS, Overall Survival; PCa, Prostate Cancer; PD, Pharmacodynamics; PFS, Progression-Free Survival; PSA, Prostate-Specific Antigen; RECIST, Response Evaluation Criteria In Solid Tumors; RP2D, Recommended Phase 2 Dose; TTR, Time To Response.

## Data Availability

All data has been included in the text.

## Abbreviations

The following abbreviations are used in this manuscript:

ABD	Antigen-Binding Domain
ADAM17	Disintegrin And Metalloproteinase 17
ADC	Antibody–drug Conjugate
ADT	Androgen Deprivation Therapy
AE	Adverse Effect
APC	Antigen Presenting Cell
BBIR	Biotin-Binding Immune Receptor
BRCA	Breast Cancer
bsAb	bispecific Antibody
CAF	Cancer-Associated Fibroblast
CAR	Chimeric Antigen Receptor
CD	Cluster of Differentiation
CID	Chemical Inducer of Dimerization
CMPC	Castration-sensitive Metastatic Prostate Cancer
CRISPR/Cas9	Clustered Regularly Interspaced Short Palindromic Repeats/Caspase 9
CRPC	Castration-Resistant Prostate Cancer
CRS	Cytokine Release Syndrome
CTC	Circulating Tumor Cell
CTLA-4	Cytotoxic T Lymphocyte-Associated protein 4
DAMP	Danger-Associated Molecular Pattern
DLT	Dose Limiting Toxicity
dMMR	Mismatch Repair deficiency
dnTGF-βRII	Dominant-Negative TGF-β Receptor II
DOR	Duration Of Response
ECM	ExtraCellular Matrix
EMT	Epithelial–Mesenchymal Transition
EpCAM	Epithelial Cell Adhesion Molecule
ERG	EST-Related Gene
GD2	disialoganglioside
GM-CSF	Granulocyte Macrophage Colony-Stimulating Factor
GPI	GlycosylPhosphatidylInositol
HD	Hinge Domain
KLK	Kallikrein
KO	knockout
FcRn	neonatal Fc Receptor
ICAM-1	InterCellular Adhesion Molecule-1
ICI	Immune Checkpoint Inhibitor
ICOS	Inducible T Cell Costimulator
IL	interleukin
iMC	inducible Costimulatory Module
ISD	Intracellular Signaling Domain
ITAM	Immunoreceptor Tyrosine-based Activation Motif
LFA-3	Leukocyte Function-associated Antigen-3
mAb	monoclonal Antibody
MAS	Macrophage Activation Syndrome
mCRPC	metastatic Castration-Resistant Prostate Cancer
MDSC	Myeloid-Derived Suppressor Cell
mPCa	metastatic Prostate Cancer
MSI-H	MicroSatellite Instability-High
MTD	Maximum Tolerated Dose;
NFAT	Nuclear Factor of Activated T cells
NKG2DL	Natural Killer Group 2 Member D Ligand
ORR	Objective Response Rate
OS	Overall Survival
PAP	Prostatic Acid Phosphatase
PCa	Prostate Cancer
PCLA	Prostate Cancer Lipid Antigen
PD	Pharmacodynamics
PD-1	Programmed cell Death protein-1
PD-L1	Programmed Death Ligand-1
PFS	Progression-Free Survival
PK	Pharmacokinetics
PSA	Prostate-Specific Antigen
PSCA	Prostate Stem Cell Antigen
PSMA	Prostate-Specific Membrane Antigen
PTEN	Phosphatase and Tensin homolog
RB	RetinoBlastoma
RECIST	Response Evaluation Criteria In Solid Tumors
RhoC	Ras homolog gene family member C
RP2D	Recommended Phase 2 Dose
SAE	Serious Adverse Event
scFv	single-chain variable Fragment
STEAP	Six-Transmembrane Epithelial Antigen of Prostate
TAA	Tumor-Associated Antigen
TACE	TNF-α converting enzyme
TAM	Tumor-Associated Macrophage
TCR	T-Cell Receptor
TGF-β	Transforming Growth Factor-beta
TIL	Tumor-Infiltrating Lymphocyte
TM	targeting molecule
TMB	Tumor Mutational Burden
TMD	TransMembrane Domain
TME	Tumor MicroEnvironment
TMPRSS2	5′-Transmembrane Protein Serine Proteinase-2
TNF	Tumor Necrosis Factor
TRAE	Treatment-Related Adverse Event
Treg	Regulatory T cell
TRUCK	T cells Redirected for Universal Cytokine Killing
T_SCM_	Stem Cell Memory T cell
TTR	Time To Response
VEGF	Vascular Endothelial Growth Factor
